# On proposing relational environmental metaphors to stimulate engagement and foster well-being in the midst of climate change

**DOI:** 10.3389/fpsyt.2024.1377205

**Published:** 2024-05-16

**Authors:** Christian R. Bellehumeur, Laure-Marie Carignan

**Affiliations:** School of Counselling, Psychotherapy and Spirituality, Faculty of Human Sciences, Saint Paul University, Ottawa, ON, Canada

**Keywords:** climate change, well-being, environmental metaphors, climate metaphors, human-environment relationship, climate engagement, Polyvagal Theory, eco-emotions

## Abstract

Messages regarding climate change that are intended to stimulate responsible engagement can impact our mental health in both positive and negative ways, which in turn can increase or limit the potential engagement being sought through those very messages. Increasingly alarmist environmental metaphors are being brought into question due to their possibly detrimental impact on mental health and well-being, and in their place, relational environmental metaphors are proffered to instill hopeful and constructive individual and collective engagement for responsible climate action. This article discusses how both alarmist and relational environmental metaphors interact with eco-emotions. It proposes, in light of concepts arising from Porges’ Polyvagal Theory − on the psychophysiology of autonomic states created in contexts of threatening cues and feelings of safety and connection −, that relational environmental metaphors are preferable for stimulating responsible collective engagement and fostering global well-being in the midst of climate change.

## Introduction

The psychological and physiological benefits of regular contact with nature for mental health and well-being, whether through green spaces (i.e. parks, forests, gardens, fields) or blue spaces (i.e. lakes, rivers, oceans) are well documented (1–3). However, climate change is disrupting that relationship (4–7). Furthermore, children and adolescents are playing less outdoors than previous generations (8, 9). They are also increasingly eco-anxious (10), in the context of media reports covering extreme events linked to climate change such as record droughts, floods, or hurricanes (11). This ecological crisis in turn leads to the use of ever more alarming metaphors (e.g. the house is burning) by politicians (e.g. 12), activists (e.g. 13) and journalists in the hopes of encouraging more eco-responsible individual and collective behaviours.

Any message regarding climate change can negatively impact the mental health of the target audience, short of including actions to support their psychological and social well-being (14). Negative psychological health is linked to a lack of positive climate engagement, or what Gifford (15) calls “dragons of inaction” (16, 17). We thus argue for the importance of constructive and hopeful, yet accurate, environmental metaphors to stimulate individual and collective engagement and foster global well-being in the midst of climate change (18: 19). Namely, we focus on the long-term merits of using relational (or ‘co-benefit’) metaphors rather than alarmist (or ‘risk reduction’) metaphors.

The purpose of this article is to provide some conceptual support as well as empirical evidence taken from previous research in order to show the benefits of using environmental and, specifically, climate-related messages drawn from metaphors that evoke positive feelings of safety, (inter)relationship and hope, as opposed to metaphors based on threat and fear (“risk reduction”) metaphors.

## Method, main sources and overview of this study

Methodologically, this study borrows an interdisciplinary perspective, drawing from various fields of research such as environmental (and climate change) communications (i.e. metaphors, 20, 21), the science of eco-emotions (22), and elements from neuroscience (via Polyvagal Theory, 23, 24). This article uses a combination of a semi-structural review (25) and a variation of a form of narrative review (22, 26). In order to do so, some guiding principles for the selection and prioritization of sources were used, such as peer-reviewed research in academic journals. There are five main parts to this article.

The first part is based on literature review, and presents some relevant research on climate metaphors. The provided selection of climate metaphors took into account space limitations. Since it would be a huge task to present all of them, and somehow impossible to make strong claims of their universality, considering the wealth of cultural diversity within multiple countries and languages, it was decided not to try to discuss all the possibilities. This article was thus partly based on a semi-structural review method recommended for such topics where many disciplines are involved and the sources differ from each other (25). In short, this article focuses on commonly encountered climate metaphors. In terms of procedural approach, database searches were made using relevant keywords such as climate + environmental + metaphors, and various related words. While not exhaustive, the main sources come from empirical studies and/or recent research published within the past ten years (19, 27–29).

The second part provides a brief overview of eco-emotions, focusing mainly on the breakthrough work of Pihkala (30). The third part presents the theoretical framework, calling upon the Polyvagal theory in particular. Taking into consideration space limitations, the fourth part presents some results from the application of the Polyvagal theory to two contrasting types of environmental metaphors (“The earth is our home” versus “Our house is burning”). Finally, in the fifth part, strengths and limitations of the study are discussed, along with some possible implications for future research.

Let us first discuss how metaphors facilitate climate change communications and campaigns before presenting recent findings on the science of eco-emotions as important drivers of climate action. Then, we will delve into Polyvagal Theory to deepen our understanding of the psychophysiology of autonomic states created in contexts of threatening cues and feelings of safety and connection (23, 24). We will discuss how feelings of safety can lead to enhanced collaborative behaviours necessary for collective engagement (23, 24), particularly in the face of climate change.

### Climate metaphors

Metaphors have been used by scientists, political leaders, environmental activists, and journalists (20, 21, 31) to communicate complex issues linked to climate change – and to trigger a response (32). Metaphors offer a way of setting the unfamiliar in familiar terms by carrying over knowledge from past experiences (20, 33). They are relevant to how we make sense of complex problems (27). Metaphors can influence people because they are more emotionally evocative than comparable literal communications (19, 34).

While a metaphorical representation facilitates understanding of certain characteristics of something, metaphors can be misleading if relied upon too heavily, in that by their very nature they create only partial insights (35). Because metaphors are not neutral ways of representing and perceiving reality, one needs to acknowledge the profound implications of commonly using a particular metaphor over another for it promotes particular readings of an event or issue (20, 36, 37). Thibodeau et al. (19) present three main ways metaphors can influence people and thus serve as effective tools of climate change communications: (1) by evoking emotions; (2) by providing a mental model for thinking (i.e. framing) the domain they describe (i.e. referring to the natural world as a complex system – say like globalisation); (3) by being accurate.

Commonly encountered environmental metaphors include ‘the house is burning’, ‘war on warming’, ‘tipping point’, and ‘greenhouse gases’, (see **Table 1** for more examples). While these metaphors convey the intended meaning and enhance comprehension of climate change (33), their anxiety-inducing nature may have unintended consequences as they impact our ability to respond. For ‘alarmist’ metaphors may reduce our ability to engage collaboratively on sustainable solutions due to neurophysiological processes involved in associative learning linking thoughts with autonomic states (24). Metaphors that rely on an alarmist or ‘risk reduction’ framework (19) may be useful in the short term to capture people’s attention, but can become detrimental by maintaining people in a state of vigilance from which they will grow fatigued (38), as they did with prolonged covid-19 precautionary measures.

**Table 1:** Commonly encountered climate metaphors and possible associated eco-emotions.

Alarmist (“risk reduction”, worrying, dichotomous, “negative”) climate metaphors	Relational (“Co-benefit”, hopeful/optimistic, constructive, “positive”) climate metaphors
War talk (“war on warming”; “fighting climate change”; “win the battle”); Tipping point(s) **Our House is Burning**; Thresholds; Sinks and Reservoirs; Greenhouse gases (Runaway greenhouse effect); Overflowing (carbon) bathtub; Heat Trapping Blanket; Loaded Dice; Natural Resources: (Bank; Store; Market; Investment); Mechanization: Spaceship; Machine; Network. Apocalypse	Mother; Parent; Child; Sister; **Our common home (The earth is our home);** Ancestor; Kingdom; Community; Gift; Anthropause
Approximative ratio of “risk reduction” metaphors versus “co-benefit” climate metaphors: 2:1	Sources: 19, 27–29
Unpleasant (“negative”, painful) eco-emotions	Pleasant (“positive”, desirable) eco-emotions
*Disappointment, Confusion;* *Shock, Trauma, Feeling Isolated;* *Fear, Worry, Anxiety, Powerlessness, Dread;* *Sadness, Grief, Yearning, Solastalgia;* *Strong Anxiety, Depression, Despair;* *Guilt, Shame, Feeling Inadequate, Regret;* *Feeling Betrayed, Disillusion, Disgust*, *Anger, Rage, Frustration;* *Hostility, Contempt, Feeling Discontent, Aversion;* *Envy, Jealousy*	*Motivation, Urge to Act, Determination;* *Pleasure, Joy, Pride;* *Hope, Optimism, Empowerment;* *Belonging, Togetherness, Connection;* *Love, Empathy, Caring, Compassion* *Admiration; Amazement, Surprise;*
Approximative Ratio “negative” versus “positive” eco-emotions: (1.7:1)	Source for all eco-emotions listed in this table: Pihkala (22)

Researchers (18, 27) have observed a tendency to overuse risk reduction metaphors, instead of more relational (hopeful/optimistic or ‘co-benefit’) ones, providing a reductionist representation of climate change and/or a separation between humans and nature – as if unrelated. The overuse of such risk reduction metaphors feeds narratives which tend to reduce the complexity of climate change and the richness of the human-nature relationship to a single dichotomous impacted/not-impacted scenario, reinforcing the economic principles of cost–benefit analysis. “This representation undermines public understanding of and engagement with climate change by marginalising subordinate policy framings which do not align with the prevailing dichotomous framing” (27, p. 34).

Although fear-based messaging is used to promote pro-ecological behaviour from the general public (39), one may wonder about its medium and long term effectiveness – especially among young people who feel more directly impacted, leading to feelings of eco-anxiety, paralysis or depression (10). How can anyone feel calm or relaxed in his/her own home while constantly being reminded that ‘the house is burning’ outside (e.g. while listening to the news)?

According to Rucińska & Fondelli (40), humans can be influenced by metaphors not only due to their intellectual ability to understand the conceptual implications (20), but also because metaphors can be defined and operationalized in more dynamic (i.e. embodied, enactive, and ecological) ways (41). For metaphors can convey many subtle and complex emotions. **Table 1** summarizes the commonly encountered climate metaphors and possible related eco-emotions in scientific literature. While not exhaustive, this list provides a brief overview which shows that “negative” climate metaphors and eco-motions are far more prominent than the “positive” ones.

### The role of eco-emotions

Interesting new advancements have recently been made in the fields of emotions studies, environmental psychology, climate emotions and eco-emotions. Works by Albrecht (42) and Pihkala (22) Pihkala (30) both acknowledge the wide scope, complexity, depth, and diversity of climate emotions, as well as the need to embrace the coexistence of their darker side (undesirable, negative, painful) and brighter side (pleasant, positive, desirable) with regards to our current natural environment. Based on their proposed typologies, both researchers propose a higher ratio for unpleasant (“negative”) versus pleasant (“positive”) eco-emotions (about 3:2 for 22; and 2:1 for 42). This seems to make sense: when things are difficult, stressful or problematic, the human mind may search for answers to explain such lack of coherence or clarity, in order to calm the state of activation (triggered by such problem) and find peace of mind (43, 44). In sum, people dealing with climate anxiety (or eco-anxiety) can experience a wide range of affects and respond differently, from high distress and even clinical symptoms (45) to finding meaning in times of adversity (46), the latter observed in terms of “practical eco-anxiety” (i.e. engaging in adapted and healthy ways despite feeling anxious about climate change, 47).

Furthermore, the study of eco-emotions is relevant to experiences of psychological resistances in the context of climate change (15). By responding to the messages conveyed by emotions − which, etymologically speaking, refer to the adaptive function of “*setting in motion”* (42)−, humans can better guide and direct behavior to better meet their needs in order to move forward. This requires a deep understanding of eco-emotions enabling us to transcend the psychological barriers of inaction (48).

Comparing various approaches for understanding social behavior and adaptive responses to stress, we can also draw parallels with climate emotions. Alarmist (risk reduction) climate metaphors (e.g. ‘our house is burning’) correspond to the classic ‘fight or flight’ response to threats (49), while “co-benefit” metaphors (e.g. ‘our common home’) resonate with the Tend and Befriend theory (50). Since the quality of attachment informs the level of safety and satisfaction within human relationships, Hlay et al. (51)’s findings suggest that secure attachments are linked to prosocial reactions to stress, namely tending to and befriending others (50).

Amidst an ongoing debate regarding the relevance of using fear-based messages versus hope-based messages in order to motivate people to adopt a more sustainable lifestyle (39), some research points in favor of using more hope-based (and/or less fear-based) messages (52–56). In short, “constructive hope” can promote pro-environmental behavior (57), policy support and political engagement (58), especially when it is understood in terms of trusting that climate change can be mitigated by solution-oriented individual and collective action (59). After all, climate messages without negative emotional content (i.e. fear-based) tend to be better received by people in general (60).

### Theoretical framework: Polyvagal Theory

Socio-cultural contexts can influence people’s ability to use as well as respond to metaphors, which both in turn impact their effectiveness (35). Furthermore, given that polyvagal theory can help shed light on various autonomic states within threatening versus safe relational contexts (23, 24, 61), we thus argue the following: this conceptual framework − which provides a neurological and physiological grounding − can support the use of positive metaphors in climate communications, particularly via climate metaphors.

In the case of relational (or “co-benefit”) environmental metaphors which may be seemingly less dire from an intellectual point of view, from the perspective of Polyvagal Theory (23, 24), such environmental metaphors may lead to greater collective involvement. Especially, if these metaphors are delivered within trusted communities and in ways that support co-regulation so as to engender feelings of safety that are maintained within individuals’ ventral vagal systems (24). Indeed, “Polyvagal Theory provides an innovative scientific perspective … of safety that incorporates an understanding of neuroanatomy and neurophysiology” (24, p. 1). Autonomic states can be regulated to promote a feeling of safety from within, which in turn leads to collaborative behaviour and social engagement (23, 24). Polyvagal Theory also validates collective involvement in promoting opportunities to experience co-regulation that contributes to feelings of safety as a reciprocal process (23, 24).

Polyvagal Theory stipulates that bidirectional pathways connect the brain’s motor cortex to the autonomic nervous system through the ventral vagal system, a “family of neural pathways” that wander (‘vagus’ is latin for ‘wanderer’) throughout the body (23). Whereas the dorsal vagal system regulates organs below the diaphragm, including the digestive system, and the sympathetic nervous system regulates blood circulation, heart rhythms and body temperature, providing energy to the system, the ventral vagal system oversees the entire autonomic nervous system, “holding the sympathetic and dorsal vagal systems in a warm embrace” (62, p.11). When the ventral vagal system is engaged as a person’s autonomic state, a feeling of safety is created.

From the perspective of Polyvagal Theory, “life is experienced from the inside out”, and these “experiences are carried in autonomic pathways” (62, p. 33). Perceptions of emotional and/or physical threats are experienced as sympathetic and dorsal vagal dysregulation, whereas feelings of safety and connection, of collaboration, creativity, and openness to possibilities are experienced in the ventral vagal autonomic state. It is important to note that in response to the same environmental context, individuals may experience differing autonomic states (24).

As Dana (62) points out, one can learn to regulate autonomic threat responses by bringing explicit awareness to previously implicit experiences. In doing so, one can interrupt the usual adaptive survival responses that typically prevent the autonomic nervous system from finding safety in connection, and thus effectively engage the “ventral vagal safety circuit” (62, p.36). Social connection is perceived as a result of ventral vagal involvement through co-regulation, but also as a “neuromodulator” that reciprocally supports the individual’s ability to maintain ventral vagal involvement, with its resulting positive effects of connection, mutual help, and cooperation (24). “Polyvagal Theory emphasizes that resilience reflects a physiological state, which is sufficiently resilient to recover from disruptions, support feelings of safety, and connect with others via an active social engagement system” (24, p. 11).

Porges (24) entertains the hope that spontaneous social engagement can emerge from communities which foster co-regulated feelings of safety, and states that “introduction of cues of safety would be a functional antidote to threat reactions by reducing the associative links between feelings of threat and thoughts and actions” (24, p.4). Furthermore, Polyvagal Theory links self-regulation and co-regulation of autonomic responses to affective experience, emotional expression, facial gestures, vocal communication, and social behavior (23). Thus, cues of safety need to be understood from the perspective of pro-social cues and social cognition.

### Applying Porges’s theory in the context of climate change: an illustration

Given the complexity and plurality of people’s emotional, behavioral and adaptive responses, according to Wright (61), Porges’ model seems useful since it provides a conceptual framework with promising heuristic potential for accounting for and/or categorizing diverse behavioral and emotional responses related to perceptions of climate change. Wright (61) suggests that in the context of climate change, people’s responses, thoughts, and feelings can be mapped following some nervous system responses. For example, social engagement responses may relate to any regulated state involving a sense of safety, curiosity, calmness, compassion and presence. However, under initial stress (or nervous activation), individuals enter into the fight or flight mode, where anger, rage or frustration are related to the fight mode, and the panic, fear, anxiety, worry, dread may echo the flight mode. Finally, feelings of hopelessness may relate to the freeze mode, and so forth (61).

Let us apply the Polyvagal Theory perspective to the two aforementioned types of opposing (i.e. alarmist/”risk reduction” versus relational/”co-benefit”) environmental metaphors. When a communicator uses a more relational metaphor such as “The earth is our home”, listeners may subjectively associate his message with the calm autonomic state which is regulated by the ventral vagal pathway, supporting homeostatic functions (i.e. health, growth, restoration) (24). This in turn activates the social engagement system which sends back signals of security (non verbal cues and facial expressions) among them and to the speaker that functionally regulate (via neuroception) autonomic states of calmness (24). Through this co-regulation, potential threat reactions can be neutralized in favor of establishing and nurturing trusting relationships (24). Furthermore, these feelings of safety not only allow sociality but can also foster problem solving and creativity, by enabling efficient access to the cortical regions (i.e. higher brain structures), optimizing health and performance (24), two important determinants of well-being (63, 64).

Another framework well-known in positive psychology may support this overall argument: the so-called “Broaden and Build theory” of positive emotions (65, 66). This theory, by adding an evolutionary dimension, may further support the psychophysiological grounding proposed by Porges (24). In brief, negative affect (emotions) narrow attentional scope, draw on existing skills and resources and arise in relation to threats which, from an evolutionary perspective, usually demand immediate attention and response (66). By contrast, positive affects (joy, pleasure, interest, etc.) broaden attentional scope, fostering the use of a wider repertoire of thoughts and actions (66). This allows for creative exploration and innovation leading to developing new skills and capabilities (such as self-regulation and emotional regulation). One may see a potential link with the ventral vagal system being the anatomical-functional instantiation of this evolved set of tendencies. Incidentally, this also echoes research on “play”, or how the creative and adaptive skills development that comes from the “playful approach” (not only for children) requires a secure, low threat “relaxed field” (67) and non-stressed frame of mind (68, p. 140).

By contrast to fostering such a secure (and thus engaging) social response via positive emotions, if someone decides to use a popular alarmist climate metaphor (i.e. “our house is burning”) with the same audience, people may feel somehow threatened, thus stressed and/or anxious, due to a subjective interpretation activating shared autonomous states of defense which disrupt homeostatic functions (24). In sum, from a psychophysiological point of view, aforementioned, we argue that the use of frequent relational (or inclusive “co-benefit”) environmental metaphors would lead to greater collective involvement if delivered within trusted communities and in ways that support co-regulation (i.e. fostering feelings of safety that are maintained within individuals’ ventral vagal systems, 24). Thus, not only the wording, but the context and manner of delivery will be important to incite greater collective engagement (24). **Table 2** summarizes this by contrasting two different environmental (or climate) metaphors using similar semantic terms, house/home. Let us underline that for most people, the notions of ‘home’ or ‘house’ refer to that of a refuge (i.e. a nocturnal symbol, 70): a place where one feels safe, can rest and enjoy life in a peaceful way, as opposed to daytime struggles linked to work, performance, achievement and so on (70).

**Table 2:** Contrasting two types of environmental (or climate) metaphors.

Elements or concepts	Types of environmental metaphors	
	“our house is burning” (12, 13)	(“the earth” is “our common home” (19, 69)
**Possible experienced meaning of the metaphor**	Alarming, need for survival and immediate protection	Belongness, common humanity
**Type of metaphors**	Dichotomous – the cause is human (CO2 effect), echoing the instrumental frame, or mechanistic frame	Relationship (‘co-benefit’) focused
**Possible triggered emotions**	Fear, anger, helplessness, alertness, sadness (loss)	Gratitude, safety, trust, joy of being together
**Hypothetical related stress behavioral responses**	Fight, flight, or freeze	Tend and befriend
**Hypothetical Attachment style**	Activated high anxiety	Secure (states of calmness allowing a higher capacity of co-regulation)
**Neural pathways**	Dorsal vagal nerve	Ventral vagal nerve

## Implications and concluding remarks

In this article, we argue that, when using environmental metaphors, we need to shift from an alarmist (or ‘risk reduction’, based on threat and fear) framework to a more relational (or ‘co-benefit’) framework (19, 71, 72) using metaphors that evoke positive feelings of safety, (inter)relationship and hope, and so forth. Based on ground work from co-emotions studies, research on stress responses, partly on Positive Psychology (i.e. Fredrickson’s model on positive emotions), and using the Polyvagal Theory, we find that metaphors focusing on climate risks may trigger debilitating fear and anxiety, especially in more vulnerable people, potentially contributing to mental health issues or prompting people to disengage (15, 53, 73).

Thus instead, and based on a more relational perspective linked to eco-emotions (74), we may pair messages acknowledging potential risks from climate hazards with messages of hope, in order to help people feel more empowered and in control of their well-being (75). To this end, we argue that metaphors offering a relational (or ‘co-benefit’) perspective rather than a ‘risk reduction’ framework such as ‘the earth is our home’ (19), or ‘our common home’ (69), may prove more successful in fostering individual and collective engagement towards global well-being in the midst of climate change (21, 71, 72). Indeed, certain metaphors favouring a human-environment relationship can lead people to adopt a more nuanced and responsible understanding of their place within the natural world (19). Metaphors grounded in a relational perspective can be viewed as more constructive and hopeful, as they favour a more systemic framework adaptation of the human-environment relationship, echoing Gillespie’s recommendation that we reframe climate change as a systemic problem (76), while focusing less on individual responsibility.

This is drawn from understanding the widespread use and psychologically influential nature of metaphors in human language and communication (e.g., the seminal works of 20), and placing it within the context of work on ‘‘eco-emotions’’ (eco-anxiety in all its forms (including ‘practical eco-anxiety’, a relatively ‘desirable’ state), depression, despair, frustration, guilt, etc.) and the tabulation of use of metaphors in climate communications. Also, by underpinning the rationale for the use of more positive metaphors by using the ‘Polyvagal Theory’ − particularly through emphasising the role of the ‘ventral vagal system’ (as opposed to the autonomic and dorsal vagal systems) and its alignment with cooperative and trusted social connection (in a reciprocal process) −, such provides a neurological and physiological grounding that supports the use of positive metaphors in climate communications.

The main limitation of this perspective paper is that it is solely conceptual (for now) and borrows various notions from diverse fields. In order to empirically support this perspective, future research may use qualitative and innovative methods to examine the same topic, such as Online photovoice (OPV) to explore people’s experiences of climate change (77–80) and Online Interpretative Phenomenological Analysis (OIPA) to understand people’s use of environmental metaphors (81).

As Marks et al. (82) have indicated, when young people can share eco-emotions and collective engagement, this can decrease their climate distress and despair, while engendering hope (83, 84), such as (‘active’ or ‘constructive’ hope; 83) which requires holding two competing realisations in mind such as: (1) the crisis is real and (2) change for a sustainable future is possible, if we keep imagining the possible while acting together now. By contrast, overuse of alarmist metaphors may notably leave youth feeling so unsafe as to stunt their ability to discover, enjoy, protect and reflect on how to engage in sustainable activity. We thus need environmental metaphors that are grounded in empirical findings yet at the same time stimulate individual and collective engagement. Such environmental metaphors may be beneficial for the current social discourse (notably inspiring political leaders), ecological education (i.e. fostering hope for future generations), and ecotherapy (i.e. helping clients deal with negative climate emotions).

## Data availability statement

The original contributions presented in the study are included in the article/supplementary material. Further inquiries can be directed to the corresponding author.

## Author contributions

CB: Writing – review & editing, Writing – original draft, Resources, Funding acquisition, Conceptualization. L-MC: Writing – review & editing, Resources.
